# The Genetic Influence on the Cortical Processing of Experimental Pain and the Moderating Effect of Pain Status

**DOI:** 10.1371/journal.pone.0013641

**Published:** 2010-10-26

**Authors:** Helen Vossen, Gunter Kenis, Bart Rutten, Jim van Os, Hermie Hermens, Richel Lousberg

**Affiliations:** 1 Department of Psychiatry and Neuropsychology, Maastricht University, Maastricht, The Netherlands; 2 Division of Psychological Medicine, London, United Kingdom; 3 Roessingh Research and Development, Enschede, The Netherlands; University of Regensburg, Germany

## Abstract

**Background:**

Research suggests that the COMT Val^158^Met, BDNF Val^66^Met and OPRM1 A^118^G polymorphisms moderate the experience of pain. In order to obtain experimental confirmation and extension of findings, cortical processing of experimentally-induced pain was used.

**Method:**

A sample of 78 individuals with chronic low back pain complaints and 37 healthy controls underwent EEG registration. Event-Related Potentials were measured in response to electrical nociceptive stimuli and moderation by COMT Val^158^Met, BDNF Val^66^Met and OPRM1 A^118^G polymorphisms was assessed.

**Results:**

Genetic variation did not have a direct effect on cortical processing of experimental pain. However, genetic effects (COMT Val^158^Met and BDNF Val^66^Met) on experimental pain were moderated by the presence of chronic pain. In the presence of chronic pain, the COMT Met allele and the BDNF Met allele augmented cortical pain processing, whilst reducing pain processing in pain-free controls. No significant effects were found concerning the OPRM1 A^118^G polymorphism.

**Conclusions:**

The current study suggests that chronic experience of pain enhances genetic sensitivity to experimentally induced mildly painful stimuli, possibly through a process of epigenetic modification.

## Introduction

The experience of pain is subject to individual differences resulting from psychological factors, behavioral factors and biological factors [1]–[4]. There is rising interest in genetic factors, as these likely explain a substantial portion of the inter-individual differences in pain responses [5]. Studies using genetically modified mice have proposed a large number of candidate ‘pain genes’[6]. In human studies, however, the list to date is much shorter. In particular, three single nucleotide polymorphisms (SNPs) have been proposed to impact on pain perception; COMT Val^158^Met (rs4680), BDNF Val^66^Met (rs6265) and the OPRM1 A^118^G (rs1799971) [7]–[9].

Catechol-O-Methyl Transferase (COMT) is an enzyme that catabolizes catecholamines and thus influences the dopaminergic and adrenergic/noradrenergic neurotransmission[10]. The COMT Val^158^Met polymorphism codes a valine (val) to metionine (met) substitution at codon 158, resulting in decreased thermostability of the COMT protein. The val158met polymorphism alters the in-vivo activity of the COMT enzyme; Val/Val homozygotes have higher levels of the COMT enzyme and correspondingly lower levels of D2 receptor neurotransmission leading to a higher level of activation of the µ-opioid system [11], [12]. On the other hand, Met/Met homozygotes have lower levels of the COMT enzyme activity, resulting in increased dopaminergic neurotransmission. COMT activity alterations associated with Val^158^Met impacts on responsiveness of the µ-opioid system, which is activated in response to pain and stressors, and typically reduces pain and response to stress [13], [14]. Met/Met homozygotes have decreased µ-opioid system activation in response to pain [15]. Therefore, Met/Met homozygotes are believed to be more sensitive to nociceptive stimuli than heterozygotes or Val/Val homozygotes.

Brain Derived Neurotrophic Factor (BDNF) is a neurotrophin that supports the growth, differentiation and survival of neurons in both the peripheral and the central nervous system. BDNF is released when nociceptors are activated and is involved in the activity-dependent pathogenesis of nociceptive pathways that may lead to chronification of pain. A genetic variation within the BDNF genes results in a valine to metionine substitution at codon 66 (Val^66^Met), resulting in reduced secretion of the BDNF protein and impaired BDNF signaling. The BDNF Val66Met polymorphism may be implicated in depression and is also hypothesized to influence pain mechanisms [16], [17]. Despite the hypothesized influence of the Val^66^Met polymorphism on pain, an online search in PubMed (http://www.ncbi.nlm.nih.gov/pubmed) with ‘BDNF Val^66^Met’ and ‘pain’ as keyword did not result in any publications.

The A^118^G polymorphism of the µ-Opioid Receptor 1(OPRM1) gene replaces adenine with guanine, increasing the receptor affinity of β-endorphin three-fold [18], [19]. It is hypothesized to increase the activity of the endogenous opioid system, which could be associated with a decreased response to nociceptive stimulation [20]. The theory that this gene polymorphism influences pain mechanisms comes mainly from mice studies reporting that opioid receptor knock-out mice had increased nociceptive responsiveness [21]. In human studies, the OPRM1 118G allele may increase the dose of morphine needed to achieve pain control [22], [23]. Furthermore, a study by Fillingim and co-workers (2005) reported that G allele carriers (infrequent allele) had significantly higher pressure pain thresholds than A allele homozygotes [18]. Lötsch and colleagues (2006) studied the influence of the G allele on cortical pain processing of experimental pain stimuli [20]. They concluded that ERP amplitudes (N1 component) of carriers of the G allele were, on average, half as high as the amplitude of the non-carriers, suggesting a lower pain processing for the G allele carriers.

The work described above yields a suggestion that several gene polymorphisms may influence pain responsiveness and that further work is required. It has been suggested that experimental designs represent a particularly powerful approach for the study of genetic effects on psychological phenotypes as they allow for controlled conditions and investigation of underlying mechanisms [24]. In pain research, Event Related Potentials (ERPs) are frequently used as a more objective measure of pain, compared to subjective pain ratings, elicited by controlled exposure to noxious stimuli [25]. Previous research has demonstrated positive correlations between the amplitude of specific peak components and the intensity of the pain stimulus, as well as between ERP amplitudes and subjective pain ratings [26]–[29]. Especially, the P1, N2 and P2 components are considered to be related to pain intensity. Thus, the aim of this study was to investigate the influence of functional variation in three gene polymorphisms on the cortical processing of experimental pain as measured with Event-Related Potentials. Results from previous studies demonstrate that the pain ERP to experimental noxious stimuli is influenced by the presence of chronic pain complaints [30]–[32]. Therefore, any influence of genetic polymorphisms was assessed in interaction with clinical pain disorder.

For every gene polymorphism two hypotheses were tested: (i) Carriers of the infrequent allele (Met allele for COMT and BDNF, and G allele for OPRM1) are expected to have different maximum peak amplitudes on the pain-specific ERP components (N1, P1, N2 and P2) in response to experimental pain stimuli compared to non-carriers [27], [33]–[35]. More specifically, concerning the COMT Val^158^Met polymorphism the Met allele carriers are expected to have larger ERP peak amplitudes compared to the non-carriers. For the BDNF Val^66^Met polymorphism, Met allele carriers are also expected to have larger amplitudes compared to the non-carriers. For the OPRM1 A^118^G, the G allele carriers are expected to have reduced amplitudes compared to non-carriers. (ii) Genetic effects are moderated by the experience of chronic pain. More specifically, we expected significant interaction effects between the polymorphisms and pain status (chronic low back pain patients versus pain free controls).

## Methods

Approval has been obtained from the medical ethics committee of the Academic Hospital Maastricht (the Netherlands), on January, 6th, 2005. All subjects gave their verbal and written informed consent prior to the experiment.

### Subjects

Two groups of subjects were included in this study. The first group consisted of 78 subjects with low back pain complaints. These subjects were drawn from the general population (via advertisements, distributed door to door) and were required to have low back pain for at least six months with no other interfering pain complaints. before the experiment. The second group consisted of 37 pain-free subjects. Exclusion criteria for both groups were the use of psychoactive drugs in general and the use of analgesics more than eight hours before the experiment. Participation was rewarded with €25,-.

### Stimuli

The stimuli used were electrical pulses of 10 ms duration, administered intracutaneously on the left middle finger. For each participant, five different intensities based on that participant's sensation and pain thresholds were administered. Of the five intensities, one was equal to the pain threshold and the other four were defined relative to this pain threshold, namely −50%, −25%, +25% and +50% of the threshold range which was defined as the range between the sensation threshold and the pain threshold. The sensation threshold was determined by first administering stimuli at zero intensity and then gradually increasing the intensity until the stimuli were experienced consciously. Once experienced consciously (sensation threshold), stimuli were once again administered with an intensity that gradually increased from the sensation threshold until the stimuli were defined as painful by the participant (pain threshold). This procedure was repeated three times in order to generate a reliable measurement.

### Rating paradigm

The stimuli were presented using a rating paradigm [36]. The paradigm consisted of 150 stimuli. The five intensities mentioned above were presented semi-randomly (Every intensity was presented 30 times in the paradigm). The inter-stimulus interval (ISI) ranged from 9 to 11 seconds. Subjects were asked to verbally rate each stimulus on a scale of 0 (no sensation) to 100 (most excruciating pain imaginable).

### EEG recording

All EEG recordings were conducted in an electrically- and sound-shielded cubicle (3*4 m^2^). Ag/AgCl electrodes were placed on Fz, Cz, Pz, C3, C4, T3 and T4 using the international 10–20 system [37]. Impedances were kept below 5 kΩ. A reference electrode was placed on each ear lobe. To control for possible vertical eye movements, an electro-oculogram (EOG) electrode was placed 1 centimetre under the midline of the right eye. A ground electrode was placed at Fpz. All electrodes were fixed using 10–20 conductive paste. Neuroscan 4.3 software was used for EEG recording.

### Procedure

Before starting the experiment, subjects were informed about the purpose of the study. Subjects were told that they would undergo EEG-registration while receiving electric shocks. After signing the informed consent form, EEG electrodes were placed and the shock electrode was attached to the top of the left middle finger as described by Bromm and Meier (1984): a small opening in the upper layer of the skin was prepared using a dental gimlet [36]. Care was taken that this procedure was not painful. In the prepared opening, a platinum electrode was placed and fixed with tape. Next, the sensation and pain threshold were determined and after that, the rating paradigm was initiated.

### Genetic analyses

Buccal cell samples were collected with sterile swabs (Omniswab, Whatman®). DNA was extracted using QIAamp DNA Mini Kits (Qiagen). In total, seven SNPs within the COMT gene were determined. The following three SNPs were genotyped using TaqMan® SNP Genotyping assays (Applied Biosystems): rs4680 (assay ID C__25746809_50), rs6265 (assay ID C__11592758_10), and rs1799971 (assay ID C___8950074_1_). All assays were run on a 7900HT Fast Real-Time PCR System (Applied Biosystems).

### ERPs

EEG was recorded with 1000 Hz sampling rate, using Neuroscan 4.3 software. Trials were selected from the continuous EEG, from 200 ms prior to the stimulus until 1500 ms post-stimulus. Data was offline filtered (bandpass 0–50 Hz) and baseline corrected. Trials with EOG activity exceeding +75mA and −75mA were excluded from the analyses.

### Statistical analyses

Multilevel random regression analyses were performed because of the hierarchical structure of the ERP data [33], [34]. The hierarchical structure of the current ERP data consist of single trials (level 1) that are clustered within individuals (level 2). This is important because trials of one person are more correlated to each other than trials for different persons. This regression technique has previously proven to be useful in the study of the ERPs [35]. The following maximum peak components, N1 (20–55 msec), P1 (56–95 msec), N2 (96–145 msec), P2 (146–300 msec), served *a priori* as dependent variable, as these components have consistently been shown to be related to the processing of stimulus intensity [27], [36]–[40]. Only ERP amplitudes of the strongest intensity (50% above the pain threshold) were used in the analyses. The N1 and P1 components are considered to be involved with the sensory processing and generated by activity in the primary and secondary somatosensory cortices. The N2 and P2 components are thought to be generated by neurons in the cingulate cortex, which is part of the limbic system which in turn is responsible for the emotional processing of pain stimuli [41]. Trial number, sensation threshold, pain threshold, age, gender, pain status and intensity of the previous stimulus served as independent variables in a basic model. Trial number was included in order to investigate, as well as correct, for difference over time (for instance habituation). It was divided in a linear effect, a quadratic and an inverse effect [35]. The sensation threshold and pain threshold were included in the model to correct for individual differences in the absolute intensities (mAmp) of the electrical stimuli that were presented. Gender and age have previously been found to influence the ERP so they were included in order to correct for their influences [42], [43]. The variable pain status refers to the chronic pain patients versus pain free controls. Finally, the intensity of the previous stimulus was included as a within-subject covariate to control for intensity sequence effect which have proven to play an influence [35]. All the independent variables, except for trial (inverse), were centered (the sample mean was subtracted from each individual score) [44].

In order to study the influences of the three gene polymorphisms they were first added (separately) as main factors to the basic model. The polymorphisms were included in the analyses as dichotomous variables (allele carriers and non-carriers). In order to test the second hypothesis, interactions between pain status and the gene polymorphisms were added.

Preliminary analyses proved that the Scaled Identity covariance structure was the best fit for our dataset. The goodness of fit (-2 Log Likelihood) of that covariance structure was significantly better than that of its competitors, namely compound symmetry (CS) and AR1. Therefore the Scaled Identity structure was used in the multilevel analyses. A random intercept was included to correct for any baseline differences between individuals. All statistical analyses were performed using SPSS 16.0. All p-values ≤0.007 (two-tailed with Bonferroni correction for the number of cranial locations, namely seven) were considered as statistically significant. Effects were considered marginally significant when p-values were between 0.050 and 0.008.

## Results

Demographic and allele/genotype distributions are displayed in table 1. Crosstabs revealed no significant difference in allelic distribution between the healthy control group and chronic pain group (COMT Met allele: p = 0.167, BDNF Met allele: p = 0.910 and OPRM1 G allele: p = 0.815). The three polymorphisms were in Hardy-Weinberg Equilibrium (χ^2^ = 1.129 p = 0.288 for COMT, χ^2^ = 0.210, p = 0.650 for BDNF and χ^2^ = 0.555, p = 0.555 for OPRM1). Furthermore, there were no significant differences in pain threshold between the Met carriers and Val homozygotes of BDNF and COMT polymorphisms and the G allele carriers and A homozygotes of the OPRM1 polymorphism.

**Table 1:** Demographic and genotypic data of the sample.

	Chronic pain group (N = 78)				Pain free controls (N = 37)			
**Demographic data**								
Gender (M/F)	38/40				16/21			
Age in years, mean (SD)	40.4 (15.4)				36.1 (14.6)			
**Experimental data**								
Sensation threshold (mV), mean (SD)	0.3 (0.1)				0.2 (0.1)*			
Pain threshold (mV), mean (SD)	1.1 (0.9)				0.8 (0.5)			
**Genotype distribution, n (%)**	**Val/Val**	**Val/Met**		**Met/Met**	**Val/Val**	**Val/Met**		**Met/Met**
COMT Val^185^Met	22 (28.6)	43 (55.8)		12 (15.6)	15 (41.7)	17 (47.2)		4 (11.1)
BDNF Val^66^Met	54 (69.2)	22 (28.2)		2 (2.6)	26 (70.2)	9 (24.3)		2 (5.5)
OPRM1 A^118^G	61 (80.3	13 (17.1)		2 (2.6)	29 (78.4)	8 (21.6)		0 (0)
**Allele distribution, n (%)**	**Carriers**		**Noncarriers**		**Carriers**		**Noncarriers**	
COMT Met allele	55 (71.4)		22 (28.6)		21 (58.3)		15 (41.7)	
BDNF Met allele	24 (30.8)		54 (69.2)		11 (29.7)		26 (70.3)	
OPRM1 G allele	15 (19.7)		61 (80.3)		8 (21.6)		29 (78.4)	

*P-value of independent sample t-test smaller than 0.05.

### The COMT Val^158^Met gene polymorphism

The first hypothesis tested for a difference in cortical pain processing between the COMT Met allele carriers and Val homozygotes. The results showed that the COMT Met allele did not directly explain variance of the pain ERP and therefore do not support the first hypothesis.

**Figure 1:**
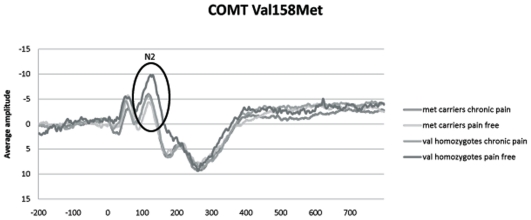
Interaction between the COMT 158Met allele and pain status. Maximum peak amplitudes of the N2-component at C4. Note that the y-axis displays negative numbers since this concerns a negative ERP component.

The second hypothesis concerned a moderation of the effect of the Met allele by chronic pain complaints (pain status). A significant interaction between pain status (chronic pain patients vs. pain-free controls) and the Met allele was found modeling the N2-component of C4 (B = −2.25, SE = 0.61, p<0.001). Furthermore, a marginally significant interaction was found at C3 (B = −1.63, SE = 0.65, p = 0.013) and T4 (B = −2.28, SE = 0.84, p = 0.008). Figure 1 illustrates the interaction effect and shows that in the pain-free control group, the COMT met carriers have lower maximum peak amplitudes (less negative) compared to the Val homozygotes. In the chronic pain group, the carriers have a higher maximum peak amplitude (more negative) compared to the Val homozygotes.

### The BDNF Val^66^Met gene polymorphism

The results for the first hypothesis did not show any direct effect of the BDNF Met allele on the pain ERP, indicating that there is no difference in cortical pain processing between Met allele carriers and Val homozygotes. Therefore the first hypothesis was not supported.

**Figure 2:**
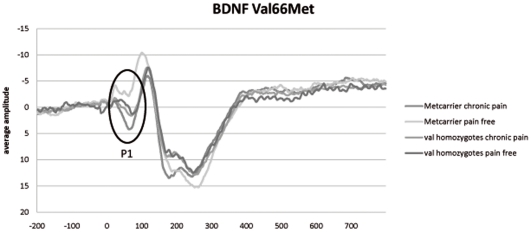
Interaction between the BDNF 66Met allele and pain status. Grand averages demonstrating the interaction between pain status and Met carriers. P1 component at Cz.

Analyses investigating the moderating effect of chronic pain complaints on the effect of the BDNF Met allele, revealed a significant interaction between pain status and the BDNF Met allele in the model of the P1- component of Fz (B = 1.87, SE = 0.63, p = 0.004), Cz (B = 1.80, SE = 0.54, p = 0.001), Pz (B = 1.34, SE = 0.46, p = 0.004, C3 (B = 1.66, SE = 0.49, p = 0.001) and a marginally significant interaction in the model of the P1-component of C4 (B = 1.40, SE = 0.55, p = 0.013). Figure 2 shows the interaction effect in the Cz models. Similar to the results of COMT, in the pain-free control group the COMT Met carriers had lower maximum peak amplitudes (less positive) than the Val homozygotes. In the chronic pain group, Met carriers had higher maximum peak amplitudes (more positive) than the Val homozygotes.

### The OPRM1 A^118^G gene polymorphism

Similar to COMT Val^158^Met and BDNF Val^66^Met, no main effect of the OPRM1 A^118^G polymorphism on the pain ERP was apparent. Carriers of the G allele thus did not respond differentially to experimental nociceptive stimuli from A homozygotes. Furthermore, no significant interactions were found for the OPRM1 genotype and pain status.

Based on the knowledge that both the COMT Val^158^Met and the OPRM1 A^118^G polymorphisms act on the mu-opioid system [45], post-hoc analyses, in which both polymorphisms were included in the model as main effects and in interaction with each other, were performed to test whether these two polymorphisms interact with each other. The results showed no moderation or mediation for these two polymorphisms. Furthermore, both COMT Val^158^Met and BDNF Val^66^Met polymorphisms have been shown to be involved in the development of depressive disorders and, there is a well known interplay between pain and depression. Post-hoc analyses revealed that individuals with chronic pain complaints reported more depressive symptoms (measured with the Beck Depression Inventory) compared to the pain free controls (µ = 6.73 (4.37) for chronic pain patients and µ = 3.02 (2.87) for pain free controls, (t-value  = −6.370, p<0.001). However, correcting the analyses for this depression effect (including the BDI score as a confounder in de multilevel regression) did not change any of the result presented in this paper.

## Discussion

The main goal of this study was to investigate the influence of three ‘pain gene candidate polymorphisms’ (COMT Val^158^Met, BDNF Val^66^Met and OPRM1 A^118^G), on pain Event Related Potentials. It was investigated whether these polymorphisms influenced the cortical processing of experimental pain and whether the presence of clinical pain influences this.

The results demonstrate that the COMT Val^158^Met polymorphism did not have a direct (main) effect on the pain ERP. In other words, independent of the covariates (intensity, trial number, sensation threshold pain status etc.), there was no difference in cortical pain processing between Met allele carriers and Val homozygotes. There was, however, an interaction between pain status and the Met allele (N2-component of C4), indicating that the effect of the COMT Met allele on the pain ERP was moderated by the presence of chronic pain complaints. When chronic pain complaints were present, COMT Met carriers displayed stronger cortical pain processing of stimuli than the Val homozygotes. When chronic pain complaints were absent, the carriers displayed weaker pain processing compared to Val homozygotes. Thus, in this study, the expected pain augmenting effect of the COMT Met allele only occurred in a group of chronic pain patients. In a pain-free population, the Met allele, if anything, may have a beneficiary effect on the cortical processing of pain.

In regard to the BDNF Val^66^Met polymorphism, the results showed no differences in cortical pain processing between Met carriers and Val homozygotes. There was, however, again a significant interaction between pain status and the Met allele at several locations. Similar to the results of the COMT polymorphism, the interaction between pain status and the BDNF Met allele indicated that when chronic low back pain complaints were present, the Met allele carriers displayed stronger ERP amplitudes (P1 component) compared to Val homozygotes. When chronic pain complaints were not present, the non-carriers displayed stronger amplitudes. Again, the hypothesized pain augmenting effect of the BDNF Met allele only occurred when chronic pain complaints were present.

The mu-opioid polymorphism OPRM1 A^118^G was the only one of the three selected polymorphisms that has previously been studied with the use of Event-Related Potentials. Lötsch and colleagues (2006) found that the G allele carriers had significantly lower N1 peak amplitude compared to the non-carriers at Cz [20]. In the present study, however, such a significant effect of the G allele was not found. This difference in result may be explained by differences in pain stimulus (nasal CO_2_ stimulation vs. electrical stimulation), stimulus duration (200 ms vs. 10 ms) and paradigm (2 intensities vs. 5 intensities). No significant interactions were found between the OPRM1 genotype and clinical status.

Thus the results demonstrate that the presence of chronic pain complaints moderates the influence of the COMT and the BDNF polymorphisms on the cortical processing of experimental pain stimuli. An explanation concerning the COMT Val^158^Met polymorphism might be alterations in endogenous opioid system that is associated with chronic pain complaints [46]. Jensen and colleagues (2009) reported that COMT Val^158^Met differences may be more expressed in individuals where the inhibitory nociceptive system is already challenged and sensitive [47]. Furthermore, it is tempting to speculate that epigenetic mechanisms play a role in this. Chronic exposures to environmental factors may result in dynamic changes of neuronal gene expression which may persist over time but can also be reversed [48]. Future research is needed to test this speculation.

The main limitation of this study most likely concerns the relatively small number of healthy controls. Therefore only the alleles and not the genotypes could be studied, precluding the study of specific recessive or dominant models. Studying genotypes could have given information about possible differences in cortical processing of pain stimuli, between the heterozygotes and homozygotes of the alleles of interest. For that reason, further research with larger populations is needed for more power. In addition stratified samples on genotype would result in an equal distribution of genotype. Also, due to the study design it cannot be entirely excluded that genetic influence on cortical processing of experimental pain may also be shaped by different affective processing between both groups. Finally, including neuroimaging tools in further research would give more detailed information on which brain areas are influenced by these polymorphisms, since Event-Related Potentials have poor spatial resolution.

The current study gives evidence that the COMT Val^158^Met and the BDNF Val^66^Met polymorphisms influence the cortical processing of experimental electrical pain stimuli, however not in a direct manner but rather under moderation of the presence of chronic pain complaints. This influence of chronic pain complaints on gene expression possibly implicates epigenetic modification.
